# Preventing ventilator-associated pneumonia: a new methodology for bed head control 24 × 7

**DOI:** 10.1186/cc10175

**Published:** 2011-06-22

**Authors:** G Vaz, RV Gonçalves

**Affiliations:** 1 Hospital Quinta D'Or, Rio de Janeiro - RJ, Brazil

## Background

Among the measures for preventing ventilator-associated pneumonia (VAP) in patients at risk, strict control of the bed head above 30° stands as the single one with better cost benefit [1]. While the semi-recumbent position is intended to be an inexpensive and easily performed action by the intensive care unit team, the smart beds currently available are not the reality for the vast majority of hospitals around the world because of the high cost. Therefore, the simple theoretical principles for its execution are contradicted by its difficult practical application.

## Objective

We propose a new methodology for continuous control of the bed head, thus making possible the appropriate compliance to the semi-recumbent position, seeking a reduction in the VAP rates.

## Methods

A retrospective observational study with 41 mechanically ventilated patients over a 7-month period starting in May 2010, in a neurointensive critical care unit of a private tertiary hospital. There was a historical control as reference during 3 months before the intervention made in August, and measurements for the same time after it as a means to confirm its appropriate implementation, based on the National Nosocomial Infections Surveillance System (NNISS) as a parameter. Applied was a technique for an hourly basis positioning of the head of bed angle in such a manner that it never remained below 30° for over 1 hour in the 24 hours daily. It was turned into a mandatory item in the prescription and its execution was performed by the nursing staff, through reading of a specific angulation marking adhesive in the side head rail, and annotation in the usual sheet for recording the vital signs, followed by the prompt adjustment to the right position. Other items of the institutional bundle of VAP were not modified.

## Results

**Figure 1:**
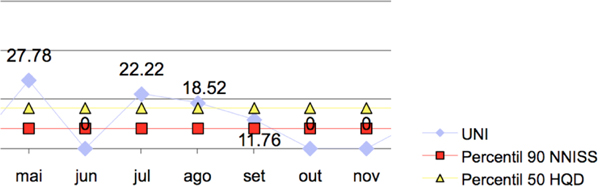
**Ventilator-associated respiratory infection rate per 1,000 days of mechanical ventilation**.

**Figure 2:**
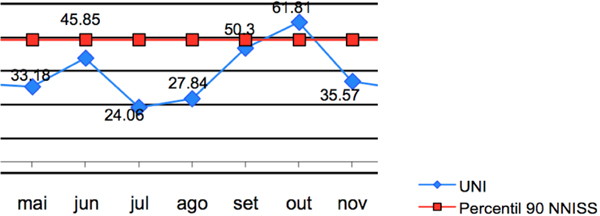
**Device utilization for mechanical ventilation**.

There was a trend towards reduction in the ventilator-associated respiratory infection rate (Figure 1) after the implementation of the methodology, bringing it to zero despite the elevation in device utilization (Figure 2).

## Conclusion

This unsophisticated and low-cost method for controlling heads of beds in an intensive care unit allowed its adequate employment, thus seeming to cause an impact in the incidence of VAP when comparing respiratory infection rate and device utilization, despite limitations about the small case series and the short following period.
